# Identify the triple-negative and non-triple-negative breast cancer by using texture features of medicale ultrasonic image: A STROBE-compliant study

**DOI:** 10.1097/MD.0000000000025878

**Published:** 2021-06-04

**Authors:** Qingyu Chen, Jianguo Xia, Jun Zhang

**Affiliations:** a Department of Ultrasonography; b Department of Radiology; c Department of Nuclear Medicine, Taizhou people's Hospital affiliated to Medical College of Yangzhou University Taizhou, China.

**Keywords:** run-length matrix, triple-negative breast cancer, ultrasound texture features

## Abstract

The study aimed to explore the value of ultrasound (US) texture analysis in the differential diagnosis of triple-negative breast cancer (TNBC) and non-TNBC.

Retrospective analysis was done on 93 patients with breast cancer (35 patients with TNBC and 38 patients with non-TNBC) who were admitted to Taizhou people's hospital from July 2015 to June 2019. All lesions were pathologically proven at surgery. US images of all patients were collected. Texture analysis of US images was performed using MaZda software package. The differences between textural features in TNBC and non-TNBC were assessed. Receiver operating characteristic curve analysis was used to compare the diagnostic performance of textural parameters showing significant difference.

Five optimal texture feature parameters were extracted from gray level run-length matrix, including gray level non-uniformity (GLNU) in horizontal direction, vertical gray level non-uniformity, GLNU in the 45 degree direction, run length non-uniformity in 135 degree direction, GLNU in the 135 degree direction. All these texture parameters were statistically higher in TNBC than in non-TNBC (*P* <.05). Receiver operating characteristic curve analysis indicated that at a threshold of 268.9068, GLNU in horizontal direction exhibited best diagnostic performance for differentiating TNBC from non-TNBC. Logistic regression model established based on all these parameters showed a sensitivity of 69.3%, specificity of 91.4% and area under the curve of 0.834.

US texture features were significantly different between TNBC and non-TNBC, US texture analysis can be used for preliminary differentiation of TNBC from non-TNBC.

## 1 Introduction

Breast cancer (BC) is 1 of the common tumors affecting the health of women in China, and there is an increase in the incidence of BC. BC is a heterogeneous disease composed of different subtypes with significant heterogeneity of morphological, genetic, and clinical characteristics. Triple-negative BC (TNBC) is a heterogeneous subtype of BC characterized by the lack of both estrogen receptor (ER), progesterone receptor (PR) and human epidermal growth factor receptor 2 (HER2).^[1]^ Tests positive for any of the three receptors is not considered as TNBC. TNBC accounts for 10% to 27% of all BC, with the highest recurrence rate and the worst prognosis.^[2–4]^

There are many methods used to diagnose BC, such as mammography, ultrasound (US), CT, and MRI.^[5–7]^ The sensitivity of X-ray mammography in the diagnosis of dense breast disease is low, and the resolution of MRI in soft tissue is higher than that of CT, but the cost is higher and the examination time is longer. Breast US is one of the most common, important and reliable methods to diagnose BC. At present, most of US studies of TNBC mainly focused on conventional US features. However, based on the diagnosis of cancer, if the pathological classification or even molecular classification of cancer can be further judged, this will provide more meaningful information to clinicians. Identifying molecular subtypes has a very important role in the diagnosis, treatment and prognosis of BC.^[8]^

Texture analysis technology can objectively provide information that cannot be observed by the naked eye and quantitatively evaluate the heterogeneity of tumors. Mazda is a software package for 2D and 3D image texture analysis, which provides a complete path for quantitative analysis of image textures, including computation of texture features/calculation of texture parameters (features), procedures for feature selection and extraction, data classification algorithms, various data visualization and image segmentation tools. Initially, MaZda was designed to analyze MRIe textures. However, MaZda has been demonstrated to be effective in analyzing other types of textured images, including X-ray, US, and CT images. Mazda has been used by researchers in different application areas, and has been proved to be an effective and reliable quantitative image analysis tool.^[9,10]^

In this study, we performed texture analysis on conventional two-dimensional US images, explored whether the texture features obtained from two-dimensional breast US images can play a role in distinguishing TNBC from non-TNBC, and investigated the value of texture analysis for the differential diagnosis of TNBC and non-TNBC, and whether the texture features can be used as an adjunct diagnostic tool in daily clinical practice.

## 2 Subjects and methods

### 2.1 Subjects

We retrospectively reviewed clinical data of 35 patients with pathologically proven TNBC who were admitted to Taizhou people's hospital from August 2015 to July 2019. And 58 patients with pathologically proven non-TNBC during the same period were included as controls. All patients were females. The mean age of patients with TNBC and non-TNBC were 57.4 ± 13.7 years (rang 36-85) and 55.8 ± 12.4 years (rang 30-90 years), respectively. Inclusion criteria were: patients who were female; had primary and unilateral single BC nodules; and did not receive neoadjuvant chemotherapy before surgery. Exclusion criteria were: patients who had bilateral BC; multifocal BC; recurrent BC; received neoadjuvant chemotherapy before surgery; and had large masses that margin was not seen completely; quality of US images was poor; and status of the HER2 amplification can not be determined by immunohistochemistry. This retrospective study was approved by the Ethics Committee of our hospital. The requirement to obtain informed consent was waived.

TNBC was diagnosed by immunohistochemistry. All samples were embedded in paraffin, SP method was used to detect the expression of ER, PR and HER-2. ER/ PR status is considered negative when the percentage of positive cells was ≤10%, negative when>10%; HER2-negative is considered if IHC result is 0(-) or 1 (+), HER2-positive is considered if IHC result is 3 (+), status of HER2 amplification need to be further determined by using fluorescence in situ hybridization (FISH) if the IHC result is 2 (+). TNBC tests negative for all three receptors.

### 2.2 US imaging

All patients underwent pre-surgical US examination using Mindray DC-8 color Doppler ultrasound system (DC-8, Mindray Bio-Medical Electronics Co., Ltd., Shenzhen, China), and the L12-3E linear array probe with a frequency of 7.5 MHz was used. Conventional US scanning was performed to observe sonographic features. Images with the clearest and most complete demonstration of lesions were chosen.

### 2.3 Image processing and texture analysis

**Figure 1:**
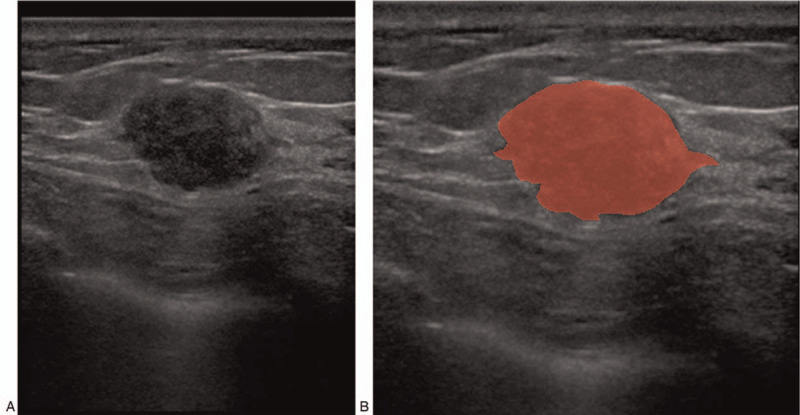
A patient with invasive ductal carcinoma of the right breast. (A) Images normalized using Matlab software (MathWorks, Inc., MA). B: ROI was manually drawn along the margins of the lesion in red.

Breast US images of all patients were exported in BMP format from PACS. First, Matlab software (MathWorks, Inc., MA) was used to normalize the images to reduce the influence of many non-constant factors related to the ultrasound scanner, such as emitted energy, brightness, contrast, reception/amplification, depth gain, noise, speckle. Then the images were imported into Mazda software (Institute of Electronics, Technical University of Lodz, Lodz, Poland, http://www.eletel.p.lodz.pl/programy/mazda/index.php?action=mazda) for delineating the region of interest (ROI) manually along the margins of tumor in red. Mazda software (Institute of Electronics, Technical University of Lodz, Lodz, Poland) can automatically generate gray level histogram, gray level co-occurrence matrix, run-length matrix, gradient model, autoregressive model, wavelet transform. ROI was manually drawn by an experienced associate chief ultrasound physician. The repeatability of manually drawing ROIs was observed, and the mean value was obtained (Fig. 1).

### 2.4 Statistical analysis

**Figure 2:**
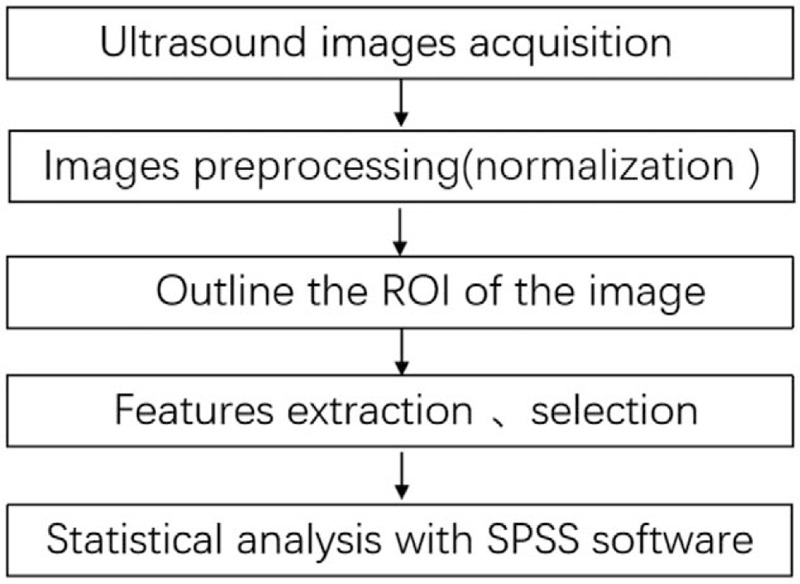
The main steps of this study.

Statistical analyses were performed using SPSS software ver. 20.0 (SPSS Inc., Chicago, IL). Variables were tested for normal distributions, normally distributed variables were compared using the two independent sample *t*-test, and non-normally distributed variables were compared using the Wilcoxon test. Quantitative data were expressed as means ± standard deviation. *P* value less than .05 was considered as significant difference. The receiver operating characteristic (ROC) curve was drawn and the area under the curve (AUC) was calculated. Multivariate logistic regression analysis was used to create model based on statistical texture parameters showing significant difference, and then the ROC curve was used to evaluate the diagnostic performance of each model. The study workflow was shown in Figure 2.

## 3 Results

A total of 93 patients with BC, including 35 patients with TNBC and 58 patients with non-TNBC were included. A total of 308 parameters were derived from gray level histogram, gray level co-occurrence matrix, run-length matrix, gradient model, autoregressive model, wavelet transform. Five optimal US image texture feature parameters were extracted from gray level run-length matrix, including gray level non-uniformity (GLNU) in horizontal direction (Horzl_GLevNonU), vertical gray level non-uniformity, GLNU in the 45 degree direction, run length non-uniformity (RLNU) in 135 degree direction, GLNU in the 135 degree direction. All these texture parameters were statistically higher in patients with TNBC compared to patients with non-TNBC (*P* < .05, Table 1).

**Table 1:** Comparison of texture parameters with statistically significant difference between patients with TNBC and non-TNBC (mean±SD).

Texture parameters	Patients with TNBC (n = 35)	Patients with non-TNBC (n = 58)	*P* value
Horzl_GLevNonU	430.74044 ± 305.7893	194.9661 ± 114.2942	<.001
Vertl_GLevNonU	475.7513 ± 365.2388	211.7448 ± 130.5161	<.001
45dgr_GLevNonU	482.9823 ± 372.4951	215.4694 ± 133.0032	<.001
135dgr_RLNonUni	15498.9211 ± 10198.0284	7807.1004 ± 4803.5708	<.001
135dgr_GLevNonU	483.1275 ± 372.7064	214.9149 ± 132.7135	<.001

**Figure 3:**
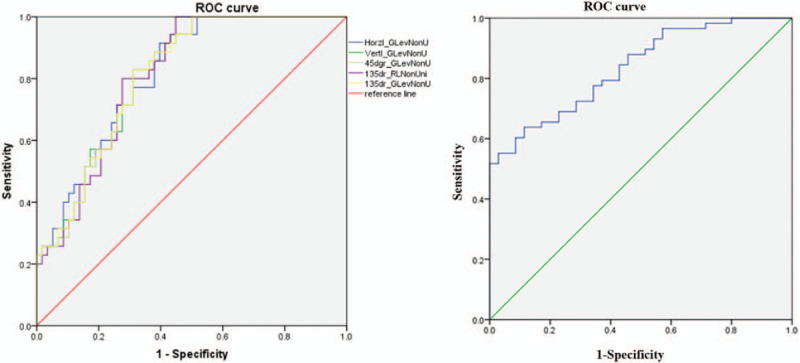
Receiver operating curves of the five optimal texture feature parameters (A) and the logistic regression analysis model established based on all these parameters (B). Horzl_GLevNonU, gray level non-uniformity (GLNU) in horizontal direction; Vertl_GLevNonU, vertical gray level non-uniformity; 45dgr_GLevNonU, GLNU in the 45 degree direction; 135dr_RLNonUni, run length non-uniformity (RLNU) in 135 degree direction; 135dr_GLevNonU, GLNU in the 135 degree direction.

Higher GLNU value represents a more non-uniform distribution of gray levels, and higher RLNU value represents a more non-uniform fineness/coarseness of the image textures. Results showed that gray level distribution and texture coarseness/fineness in patients with TNBC were more non-uniform compared with patients with non-TNBC. With the use of ROC curve analysis, the thresholds, sensitivity, specificity and AUCs for texture parameters that were significantly different were calculated (Table 2, Fig. 3A). The results revealed that at a threshold of 268.9068, Horzl_GLevNonU showed the best diagnostic performance in differentiating TNBC from non-TNBC. The logistic regression model established from combining all five texture parameters that were significantly different yielded a sensitivity of 69.3%, specificity of 91.4% and AUC of 0.834 (Table 2, Fig. 3B).

**Table 2:** Diagnostic performance of texture parameters with statistically significant difference between patients with TNBC and non-TNBC.

Texture parameters	Threshold	Sensitivity	Specificity	AUC	95%CI
Horzl_GLevNonU	268.9068	71.4	74.1	0.814	0.731∼0.898
Vertl_GLevNonU	233.3091	88.6	62.1	0.811	0.727∼0.895
45dgr_GLevNonU	294.9278	71.4	72.4	0.809	0.725∼0.894
135dr_RLNonUni	9555.2776	80	72.4	0.809	0.725∼0.894
135dr_GLevNonU	244.1432	85.7	63.8	0.810	0.726∼0.895
Combinations of texture parameters	0.7249	69.3	91.4	0.834	0.756∼0.913

## 4 Discussion

TNBC is the most aggressive BC subtype that stains negatively for ER, PR and HER2 during immunohistochemistry. Clinical features of TNBC include rapid disease progression, high metastasis, earlier onset age, and high mortality. There are no targeted drugs to treat TNBC. Neoadjuvant chemotherapy before surgery can significantly extend the survival time of patients.^[11]^ Therefore, early and accurate diagnosis is essential for treatment of TNBC. Regarding the early diagnosis of TNBC, previous study showed that values of perfusion parameters on contrast-enhanced US were significantly greater in estrogen receptor-negative tumors than that in estrogen receptor-positive tumors, indicating that perfusion parameters on contrast-enhanced US have good predictive value for estrogen receptor-negative tumors.^[12]^ Çelebi et al^[13]^ found that US characteristics are closely associated with the molecular subtype, histological grade and hormone receptor status of the tumor, tumors showing posterior attenuating were more often non-triple negative subtype, and tumors with circumscribed margins were more often triple-negative subtype. Krizmanich-Conniff et al^[14]^ indicated that TNBC frequently manifested as an irregular, non-calcified mass with ill-defined or spiculated margins on mammography. A retrospective study reported by Jin et al^[15]^ found that MRI examination can be used for preliminary prediction of molecular subtypes of BC before surgery, however, there is a risk of allergic reaction due to the injection of contrast media, using MRI may result in economic burden to patients. Interpreting mammograms is highly subjective. Compared with the above examinations, diagnosis of BC with US texture analysis do not rely on the personal experiences of physicians, and can extract more objective texture information that cannot be recognized by naked eye, thereby achieving the purpose of assisting doctors in diagnosing TNBC.

Using texture analysis of CT, MRI and US images to assist diagnosis is the current research hotspot.^[16,17]^ However, the value of US texture analysis for the differential diagnosis of TNBC from non-TNBC is rarely reported, therefore, in this study we explored this issue. All images are composed of different microscopic primitives, when regular or random repetition of the primitives occurs, image structural texture is formed. Texture features are important source of information for understanding images. Texture features can also be extracted from US images, the ability of human eye to distinguish gray level and spatial distribution in texture is limited. Computer image texture analysis technology can provide a wealth of subtle information invisible to the human eye, that holds promise for the quantitative analysis of US image textures. US texture analysis can be used to analyze the relationship between texture feature values and tumor pathological types, as well as the relationship between texture parameters and tissue status. To hope that the parameters reflecting tissue status can be isolated from US image textures in order to differentiate different specific BC subtypes.

Previous studies have found that due to the high degree of malignancy and rapid growth of TNBC, the growth inside the tumor varies, and although tumor microvessel density is high, but the distribution of microvessels was uneven.^[18,19]^ In this study, we compared the texture features of US images between patients with TNBC and non-TNBC using Mazda software, the results showed that several texture parameters based on gray level histogram, gray level co-occurrence matrix, run-length matrix, gradient model, autoregressive model, wavelet transform had positive significant effect. Five texture parameters from run-length matrix, including Horzl_GLevNonU, vertical gray level non-uniformity, X GLNU in the 45 degree direction, RLNU in 135 degree direction, GLNU in the 135 degree direction, showed significant difference between patients with TNBC and non-TNBC, texture parameter values were significantly higher in patients with TNBC compared to patients with non-TNBC (*P* < .001). The run-length matrix is a second order statistically method. Higher GLNU value indicates a more non-uniform gray-level distribution. This may be due to cellular necrosis and the lack of fibrous tissue caused by uneven blood flow within the mass of patients with TNBC. The results also indicated that the tumor tissue in patients with TNBC is poorly differentiated, with significantly higher degree of malignancy compared with patients with non-TNBC. Lee et al^[2]^ found that US texure analysis presented a high diagnostic performance in the differential diagnosis of fibroadenoma and TNBC, even in BI-RADS category 3 and 4a lesions. Zhang et al^[5]^ found that the texure features help to distinguish TNBC from non-TNBC and are associated with breast tumor histology. There is a certain association between the biological heterogeneity of TNBC and the heterogeneity of US textures. Previous studies have found that under both same and different conditions, run-length matrix parameters computed from the same area of the same tumor mass are measurable and repeatable, run-length matrix parameters computed from different tumor masses can be compared,^[20–22]^ which further supported the credibility of the results of this study.

In this study, we used US image texture analysis for differential diagnosis of TNBC from non-TNBC, the greatest advantages of this method include non-invasive, risk-free, and this method does not increase the economic burden of patients. Texture analyses of US image has been shown to have potential for differential diagnosis of BC subtypes, and it opens up new avenues for non-invasive diagnosis.^[2,23]^ Contrast-enhanced ultrasonography and MRI have high value for evaluation of heterogeneity of BC, but there is a risk of allergic reaction to contrast media. In addition, MRI examination results a certain economic burden to patients. Compared with conventional imaging, image texture analysis does not rely on the personal experience of physicians, can extract important texture information that cannot be recognized by the human eye, and analyze gray level distribution of pixels to reflect the lesion heterogeneity, thereby achieving the purpose of quantitative diagnosis, classification, gene expression analysis, and prognosis evaluation of the disease.

We acknowledge the following limitations in our study: Firstly, The sample size is relatively small, images obtained by the same instrument were selected, certain selection bias may occur, and we will expand the sample size in the future to confirm the findings. Secondly, retrospective study based on US texture features cannot completely overcome the operator dependency of the initial examination. Finally, we only included patients with TNBC and non-TNBC, the differences in texture features in pathological subtypes of BC can be analyzed in the future.

In conclusion, US examination has the advantages of low cost and no radiation exposure, which is currently widely used in clinical diagnosis.^[24,25]^ With the widespread use of US imaging equipment, better image processing techniques are needed for doctors for accurate diagnosis of disease. Texture analysis can capture subtle changes in pixels of US images. US texture analysis can provide an objective adjunct diagnostic tool and improves the diagnostic efficiency for the differential diagnosis of TNBC from non-TNBC, reduce the number of invasive biopsies, and provide evidences for the clinic clinical diagnosis and treatment.

## Author contributions

**Conceptualization:** Jun Zhang.

**Data curation:** Qingyu Chen, Jianguo Xia.

**Formal analysis:** Qingyu Chen, Jianguo Xia, Jun Zhang.

**Investigation:** Qingyu Chen, Jun Zhang.

**Methodology:** Jun Zhang.

**Resources:** Jianguo Xia.

**Software:** Jianguo Xia.

**Writing – original draft:** Qingyu Chen.

**Writing – review & editing:** Jianguo Xia, Jun Zhang.
